# Clinical Evaluation of the Peroral Cholangioscopy Using a New Videoscope

**DOI:** 10.1155/DTE.5.231

**Published:** 1999

**Authors:** Yoshinori Igarashi, Takeo Ukita, Hirokazu Inoue, Jun Ishiguro, Satoshi Ogawa, Masahiro Satou, Iruru Maetani, Yoshihiro Sakai

**Affiliations:** Third Department of Internal Medicine Division of Digestive Endoscopy Ohashi Hospital Toho University School of Medicine, 2-17-6 Ohashi, Meguro-ku Tokyo Japan

## Abstract

Peroral cholangioscopy (PCS) has been performed in 22 cases using XCHF-B200 (Olympus Optical Co.) since June 1995 and in 77 cases using CHF-B20 (Olympus Optical Co.) after EST from Jan. 1989. XCHF-B200 has a longer rigid portion of distal end and a smaller channel diameter than CHF-B20. The successful rate of PCS using XCHF-B200 (82%) was lower than that of CHF-B20 (89%). The vascular pattern and fine vertical groove of the bile duct mucosa were shown more clearly on the photographs obtained with XCHF-B200 than those obtained with CHF-B20. However, not enough biopsy specimens could be obtained because the channel diameter of XCHF-B200 was rather small.

If the length of rigid portion and biopsy channel of XCHF-B200 are improved, PCS using XCHF-B200 will be more useful for the diagnosis of bile duct disorders.


*Diagnostic and Therapeutic Endoscopy*, Vol. 5, pp. 231–237
Reprints available directly from the publisher
Photocopying permitted by license only

© 1999 OPA (Overseas Publishers Association) N.V.
Published by license under
the Harwood Academic Publishers imprint,
part of The Gordon and Breach Publishing Group.
Printed in Malaysia.

### Clinical Evaluation of the Peroral Cholangioscopy using a new Videoscope

YOSHINORI IGARASHI*, TAKEO UKITA, HIROKAZU INOUE, JUN ISHIGURO, SATOSHI OGAWA, MASAHIRO SATOU, IRURU MAETANI and YOSHIHIRO SAKAI

*Third Department of Internal Medicine, Division of Digestive Endoscopy, Ohashi Hospital, Toho University School of Medicine, 2-17-6 Ohashi, Meguro-ku, Tokyo, Japan*

*(Received 11 September 1998; Revised 5 January 1999; In final form 5 March 1999)*

**Peroral cholangioscopy (PCS) has been performed in 22 cases using XCHF-B200 (Olympus Optical Co.) since June 1995 and in 77 cases using CHF-B20 (Olympus Optical Co.) after EST from Jan. 1989. XCHF-B200 has a longer rigid portion of distal end and a smaller channel diameter than CHF-B20. The successful rate of PCS using XCHF-B200 (82%) was lower than that of CHF-B20 (89%). The vascular pattern and fine vertical groove of the bile duct mucosa were shown more clearly on the photographs obtained with XCHF-B200 than those obtained with CHF-B20. However, not enough biopsy specimens could be obtained because the channel diameter of XCHF-B200 was rather small.**

**If the length of rigid portion and biopsy channel of XCHF-B200 are improved, PCS using XCHF-B200 will be more useful for the diagnosis of bile duct disorders.**

*Keywords:* Cholangioscopy, Electronic endoscopy, Endoscopy, Peroral cholangioscopy

### BACKGROUND

Electronic endoscopy was introduced for the clinical use against the digestive tract disorder in 1984 [1–3]. It has been shown to be useful for the diagnosis of small and flat mucosal lesions. However little improvement in the cholangioscope itself has been made. In particular, using peroral cholangioscopy was clinically difficult, because the scope was too large to pass through the main papilla.

With the development of equipment-manufacturing technology including the small sized charge-coupled device (CCD) in recent years, however, a new electronic cholangioscope has been developed (XCHF-B200, Olympus Optical Co.) [4]. We compared an ordinary CHF-B20 with a new XCHF-B200 as to the diagnostic ability. This paper is a report of our findings.

* Corresponding author. Tel.: 0081-3-3468-1251. Fax: 0081-3-3468-1269.

### MATERIALS AND METHODS

Peroral cholangioscopy (PCS) using XCHF-B200 was performed in 22 cases during a period from June 1995 to Dec. 1997: every case had received endoscopic sphincterotomy (EST) (choledocholithiasis in 21 cases and cholangioma in 1 case). PCS using CHF-B20 was performed in 77 cases during a period from Jan. 1989 to Dec. 1997. All cases with choledocholithiasis received endoscopic treatments. In 8 cases, PCS using both models was performed for taking biopsy specimens. The dye (5% methyleneblue [5]) was administered through the biopsy channel in 4 of 8 cases in which both XCHF-B200 (Fig. 1) and CHF-B20 were used. All the patients agreed to participate in our study and gave their informed consent.

Before PCS was started, topical pharyngeal anesthesia (lidocaine, 2% gel), intravenous administration of diazepam (10 mg) and scopolamine butylbromide (20 mg) were given. As a mother scope, TJF-M20 (Olympus Optical Co.) was used.

According to the specifications of the XCHF-B200 (Table I), the length of rigid portion is about 5 mm longer than that of CHF-B20. The channel diameter of XCHF-B200 is 1.2 mm, and smaller than that of CHF-B20. Therefore the biopsy forceps (5 Fr) cannot be used to take a biopsy specimen. But thinner basket forceps (1.0 mm) were used to remove a small stone (Fig. 2(a) and (b)). XCHF-B200 has a magnifying (× 2.6 times) and a contour-emphasizing systems. Endoscopic photographs were taken using the EVIS-200 system (Olympus Optical Co.).

We compared the success rate of PCS with that of XCHF-B200 and CHF-B20. Endoscopic pictures of both PCS were studied for the upper part of the common bile duct mucosa which was less influenced by insertion of the scope. The endoscopists (experience over 10 years) judged endoscopic photographs and VTR.

In 18 XCHF-B200 cases, we evaluated endoscopic pictures of the vascular pattern (Fig. 3) and

TABLE I Specifications of peroral cholangioscope

| | CHF-B20 | XCHF-B200 |
|---|---|---|
| Angle of view | 100° | 100° |
| Depth of view | 3 ~ 50 mm | 3 ~ 50 mm |
| Outer diameter (Distal end) | 4.1 mm | 4.1 mm |
| Outer diameter (Insertion tube) | 4.5 mm | 4.5 mm |
| Rigid portion (Distal end) | 9 mm* | 14 mm* |
| Max. bending angle | 160°/100° | 160°/100° |
| Working length | 1870 mm | 1870 mm |
| Total length | 2190 mm | 2190 mm |
| Channel diameter | 1.7 mm | 1.2 mm |
| Mother scope | TJF-M20 | TJF-M20 |

*This value was measured by myself.

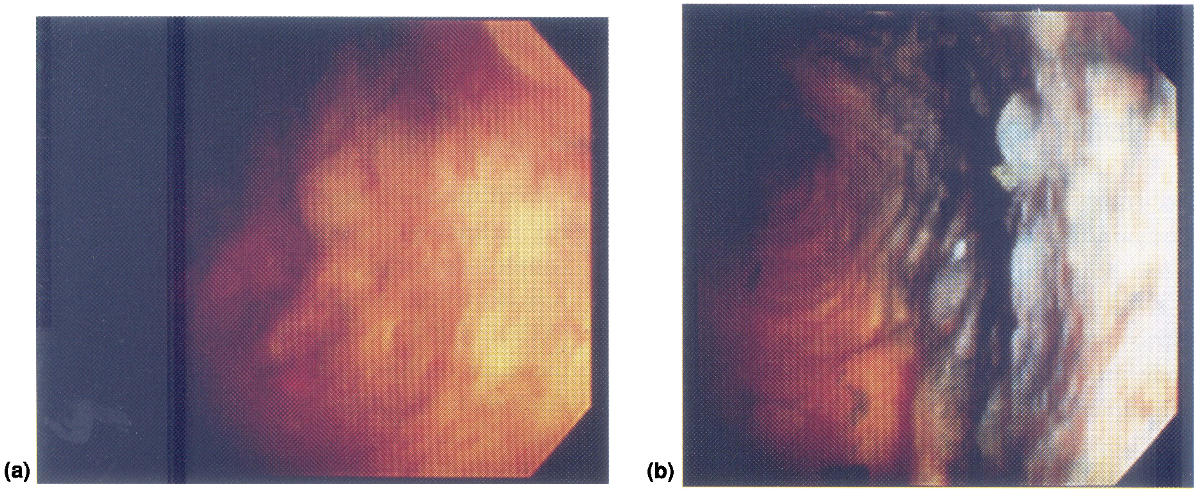

FIGURE 1 (a) Endoscopic picture showing the uneven mucosa of the bile duct. (b) Endoscopic picture showing that the uneven mucosa is clearer after dyeing.

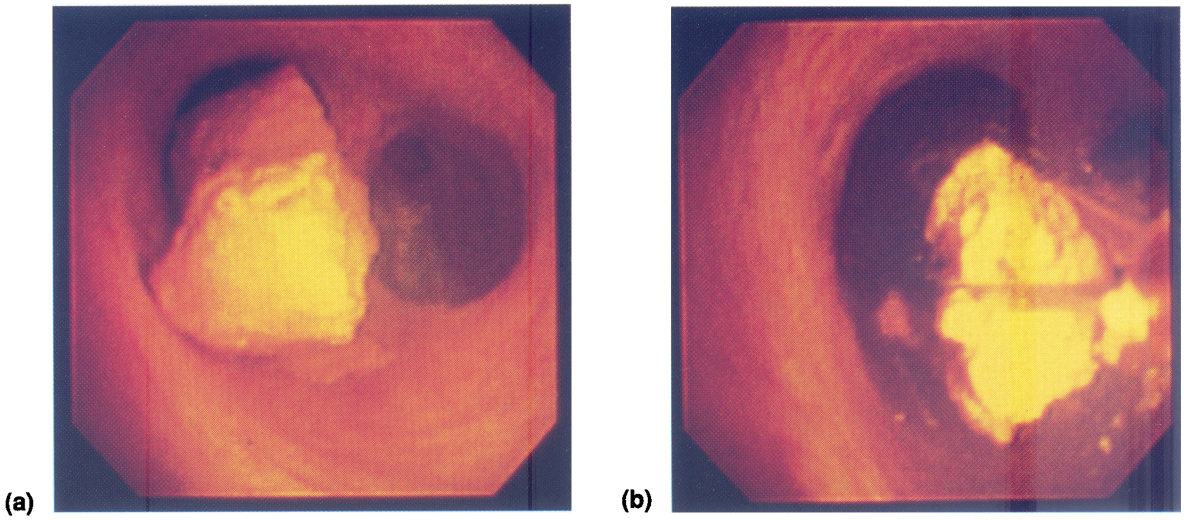

FIGURE 2 (a) Endoscopic picture showing choledocholithiasis in the common bile duct. (b) Endoscopic picture showing the stone extraction using a thinner basket catheter under XCHF-B200.

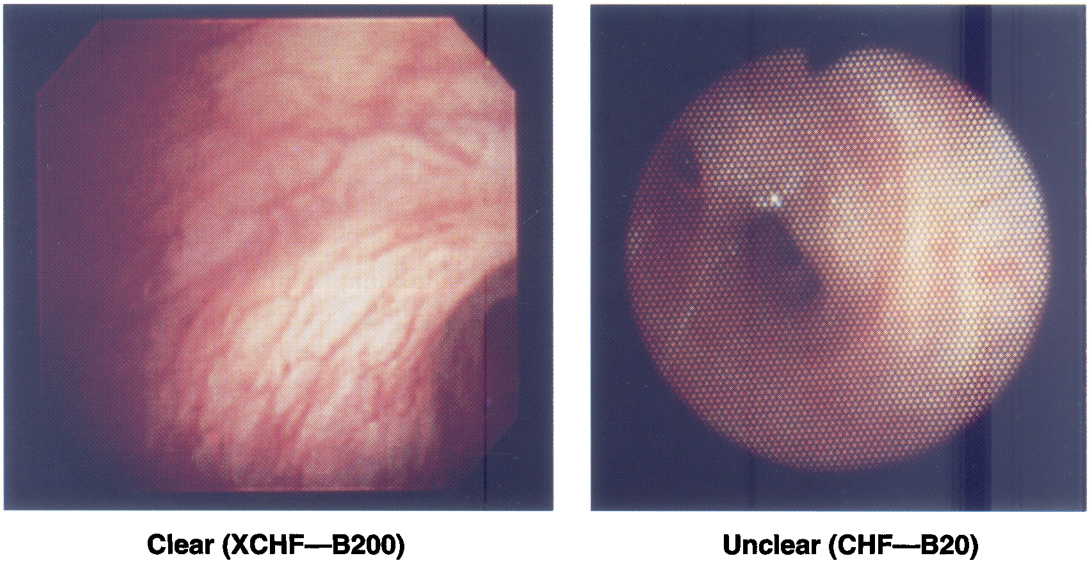

FIGURE 3 Endoscopic picture using XCHF-B200 showing the clearly vascular pattern of the bile duct mucosa. Endoscopic picture using CHF-B20 is unclear.

fine vertical groove (Fig. 4) in the bile duct mucosa. In 8 cases, we compared the endoscopic pictures of XCHF-B200 and CHF-B20. When the observation with XCHF-B200 revealed some lesions, we changed XCHF-B200 to CHF-B20 for taking biopsy specimens. Comparison of both groups was made by the chi-square test. The statistical significance was defined as $p < 0.05$.

### RESULTS

PCS using XCHF-B200 was successful in 18 cases (82%). In 4 cases, it failed because XCHF-B200 could not pass through the small EST site (Table II). In 18 cases, the videoscope was successfully inserted into the bilateral hepatic ducts. In one case, it could be inserted into the neck of the gallbladder after

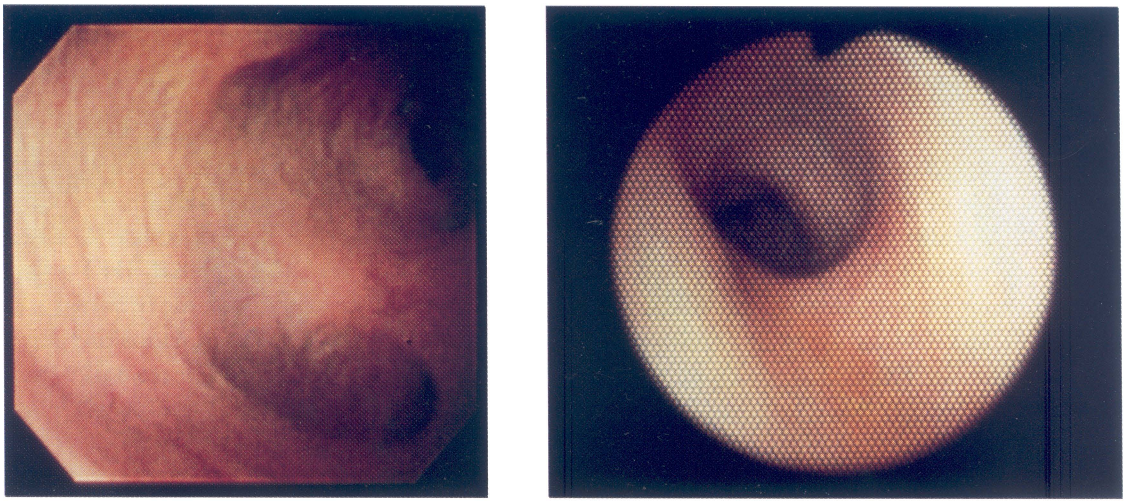

FIGURE 4 Endoscopic picture using XCHF-B200 showing the fine vertical groove of the bile duct mucosa. But endoscopic picture using CHF-B20 is unclear.

TABLE II A success rate of the introduction into the common bile duct

| | Successful rate | |
|---|---|---|
| XCHF-B200 ($n=22$) | 18 (82%) | NS |
| CHF-B20 ($n=77$) | 68 (89%) | |

Reason for failure: no passage through the EST site in all cases.

endoscopic lithotomy for the impacted stones in the cystic duct (Fig. 5). PCS using CHF-B20 was successful in 68 cases (89%). In 9 cases, it failed by the same reasons as with XCHF-B200.

Endoscopic pictures obtained with XCHF-B200 showed clear vascular patterns of the bile duct mucosa in all of the 18 cases and fine vertical groove in 15 cases (83%) (Table III). In 8 cases in which both models were used, vascular patterns obtained with XCHF-B200 were clearer than those obtained with CHF-B20 (Table IV). The fine vertical groove was present in 7 cases with the images obtained with XCHF-B200 being clearer than those obtained with CHF-B20 (Table V). After dyeing endoscopic images became clearer with XCHF-B200 (Table VI, Fig. 1). In 3 cases in which CHF-B20 was used, images were unclear after dyeing because the light intensity was insufficient. This procedure was performed safely in all the cases without any complications.

### DISCUSSION

Various techniques for PCS have been used clinicaliy [6,7]. Recently, PCS by the mother–baby scope technique has become popular. Many types of baby scopes [8–10] have been developed. CHF-B20 functions are well for diagnosis of lesions in the intrahepatic duct, common bile duct, cystic duct [11] and the gallbladder [12]. An ultra-small fiberscope having an external diameter [13] of 0.8 mm is available for insertion into the gallbladder without EST, but the light intensity obtained is insufficient. PCS using a miniscope (2.09 mm) [14] and the guide-catheter method [15] has been performed without EST, but the biopsy forceps could not be used. For the cholangioscope with a biopsy channel, it is necsssary to perform EST before PCS. Evenwhere EST has been performed before treatment, the use of the cholangioscope is limited because of its small orifice size. The light intensity of cholangioscopes is also limited by the scope size. Therefore, the

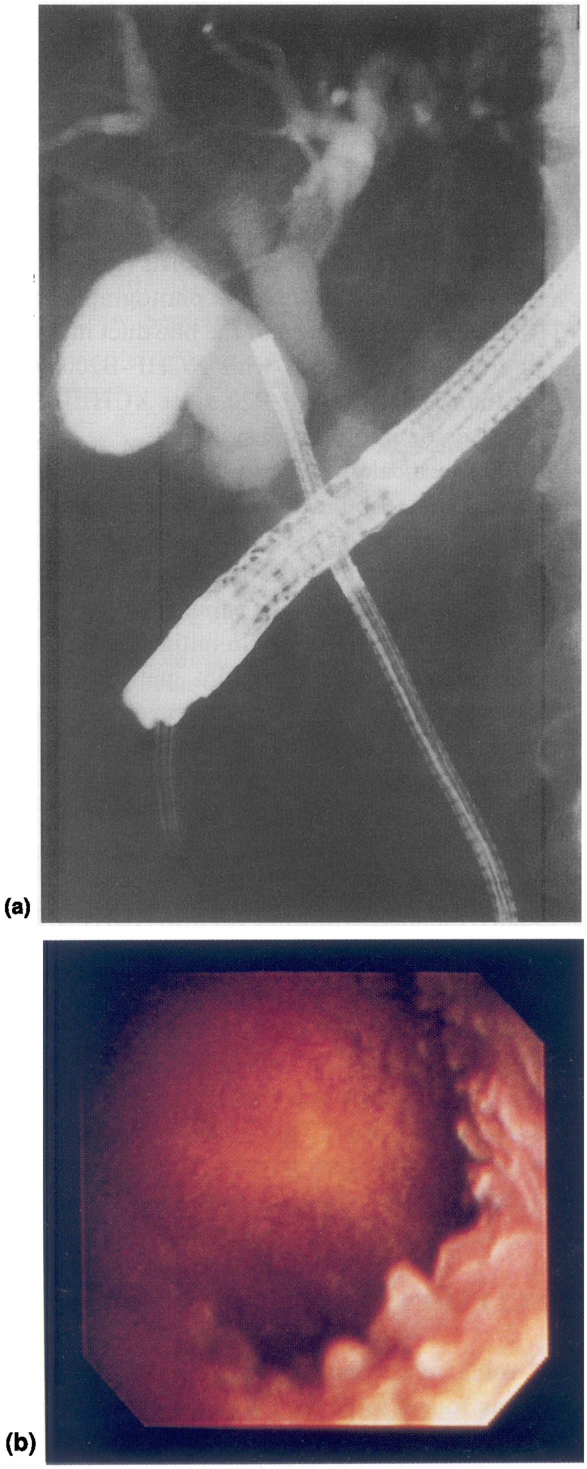

FIGURE 5 (a) ERC findings showing the XCHF-B200 inserted in the neck of the gallbladder. (b) Endoscopic picture showing the mucosa of the gallbladder.

TABLE III Visualization of vascular pattern and fine vertical groove of the bile duct mucosa on photographs

| | Clear | Unclear |
|---|---|---|
| Vascular pattern | 18 | 0 |
| Fine vertical groove | 15 | 3 |

1995.7–1998.12.

TABLE IV Visualization of vascular pattern of the bile duct mucosa at eight cases on photographs

| | Clear | Unclear | |
|---|---|---|---|
| XCHF-B200 | 8 | 0 | * |
| CHF-B20 | 3 | 5 | |

*$p < 0.05$.

TABLE V Visualization of fine vertical groove of the bile duct mucosa at eight cases on photographs

| | Clear | Unclear | |
|---|---|---|---|
| XCHF-B200 | 7 | 1 | * |
| CHF-B20 | 3 | 5 | |

*$p < 0.05$.

TABLE VI Visualization of fine mucosal pattern after surface dyeing on photographs

| | Clear | Unclear | |
|---|---|---|---|
| XCHF-B200 ($n = 4$) | 4 | 0 | NS |
| CHF-B20 ($n = 4$) | 1 | 3 | |

endoscopic pictures obtained with cholangioscopes are sometimes insufficient due to poor light intensity. We took endoscopic pictures carefully with a cholangioscope and recorded them on a videoscope.

In 1984, an electronic endoscope was clinically introduced to observe the digestive tract lesions [1–3]. The diagnostic ability of the electronic endoscope to detect superficial and minute lesions of early cancer has since been improved. However, because the tip of the electronic endoscope had to contain a large CCD, the instrument was bigger than the EST site. It could not be passed through there so that a clinical trial of this instrument for the biliary tract was postponed.

Now, the size of CCD has been made smaller and percutaneous transhepatic cholangioscopy (PTCS) can be performed under an electronic cholangioscope [16]. This has improved diagnosis of biliary tract diseases. However, the electronic cholangioscope using PTCS has an external diameter of 5.3 mm and requires an 18 Fr fistula. Scopes over 5 mm in diameter cannot be used for PCS, and cannot pass through the main papilla after EST.

With the size of CCD reduced, electronic cholangioscopes have become smaller in outer diameter. Now PCS can be performed with an electronic cholangioscope (XCHF-B200) [4]. XCHF-B200 (4.5 mm) with a CCD built in has a longer rigid portion of distal end (+5 mm) than CHF-B20 (Fig. 6). The channel diameter of XCHF-B200 is smaller than that of CHF-B20. The success rate of PCS using XCHF-B200 (82%) was correspondingly lower than that with CHF-B20 (89%), but the difference was not significant. The long rigid portion of distal end of XCHF-B200 is more difficult to pass through the EST site.

Pancreatoscopy with XCHF-B200 is very difficult because the main pancreatic duct usually has a diameter of less than 3 mm in diameter. Mucin-producing tumors occasionally showed a dilated pancreatic duct [4]. XCHF-B200 can be used only in patients with such tumors.

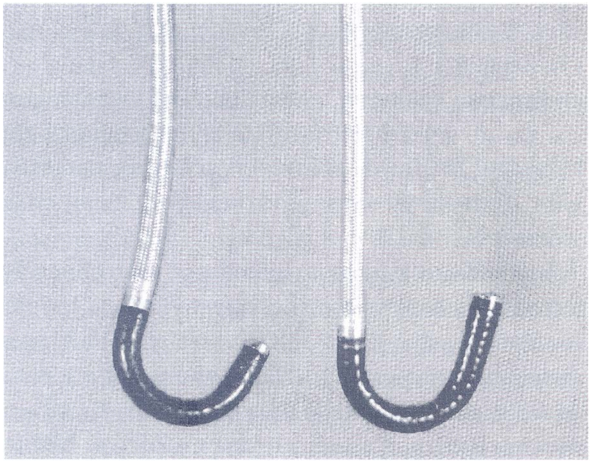

**CHF—B20** **XCHF—B200**

FIGURE 6 Two endoscopes showing side by side. The rigid portion of distal end of XCHF-B200 is about 5 mm longer than that of CHF-B20.

When electronic endoscopes are used for PCS, emphatic contours and color tones can be obtained. We compared endoscopic pictures obtained with XCHF-B200 to those obtained with CHF-B20. This study used a small number of cases, but it is able to judge objectively by three endoscopists (experience over 10 years) using endoscopic photographs and VTR. The vascular pattern of the bile duct mucosa on photographs obtained with XCHF-B200 was clearer than that with CHF-B20, since XCHF-B200 uses a red color emphasizing system.

The fine vertical groove of the bile duct mucosa on photographs obtained with XCHF-B200 was clearer than that with CHF-B20. We think that it is very useful for diagnosing especially early carcinoma of the bile duct because papillary and granularmucosa of the bile duct is an initial marker [17]. When endoscopic dyeing with 5% methylene blue was performed under CHF-B20, good images were not obtained, because the light intensity was insufficient. However endoscopic images by XCHF-B200 were clear even after dyeing. It is useful for diagnosing the spread of cholangiocarcinoma [5].

Finally, the success rate of PCS with XCHF-B200 was lower than that with CHF-B20. The rigid portion of distal end of XCHF-B200 is longer than that of CHF-B20. However, endoscopic images with XCHF-B200 were clearer than those with CHF-B20. The problem is that the biopsy channel of XCHF-B200 being 1.2 mm in diameter, does not allow the biopsy forceps to pass through it, which precludes histological diagnosis.

### CONCLUSION

PCS using electronic cholangioscope XCHF-B200 was found to be very useful for diagnosing the biliary tract diseases. In particular, endoscopic images obtained with XCHF-B200 were clearer than those obtained with CHF-B20. When the electronic system and biopsy channel of XCHF-B200

are improved, PCS with XCHF-B200 will be more useful for diagnosing bile duct diseases.

### *Acknowledgment*

The major findings of this study were reported at The 51st Congress of the Japan Gastroenterological Endoscopy Society, April 18, 1996, Kobe, Japan and The 7th Asian-Pacific Congress of Digestive Endoscopy, September, 1996, Yokohama, Japan.

### *References*

[1] Classen, M. and Phillip, J. Electronic endoscopy of the gastrointestinal tract. Initial experience with a new type of endoscope that has no fiberoptic bundle for imaging. *Endoscopy* 1984; **16**: 16–19.

[2] Matek, W., Lux, G., Riemann, J.F. *et al.* Initial experience with the new electronic endoscope. *Endoscopy* 1984; **16**: 20–21.

[3] Sivak, M.V. Video endoscopy. *Clin. Gastroenterology* 1986; **15**: 205–234.

[4] Tanaka, K., Mukai, H., Nakajima, M. *et al.* Clinical evaluation of an electronic endoscope system for peroral cholangiopancreatoscopy (PCPS). *Gastroenterol. Endosc.* 1998; **40**: 824–832 (in Japanese with English abstract).

[5] Nishikawa, K., Ogawa, S., Sato, M. *et al.* A study of malignant bile duct stenosis using percutaneous transhepatic cholangioscopy. *Dig. Endoscopy* 1991; **3**: 342–349.

[6] Takekoshi, T. and Takagi, K. Retrograde pancreatocholangioscopy. *Gastroenterol. Endosc.* 1975; **17**: 678–683 (in Japanese with English abstract).

[7] Rosch, W., Koch, H. and Demling, L. Reports on new instruments and new methods. *Endoscopy* 1976; **24**: 172–174.

[8] Bar-Meir, S. and Rotmensch, S. A comparison between peroral choledochoscopy and endoscopic retrograde cholangiopancreattography. *Gastrointestinal Endosc.* 1987; **1**: 13–14.

[9] Kozarek, R.A. Direct cholangioscopy and pancreatoscopy at time of endoscopic retrograde cholanglopancreatography. *Am. J. Gastroenterol.* 1988; **83**: 55–57.

[10] Riemann, J.F., Kohler, B., Harloff, M. *et al.* Peroral cholangioscopy – an improved method in the diagnosis of common bile duct diseases. *Gastrointestinal Endosc.* 1989; **35**: 435–437.

[11] Igarashi, Y., Katagiri, K., Kishi, H. *et al.* An endoscopic study of the cystic duct under peroral cholangioscopy. *Gastroenterol. Endosc.* 1992; **34**: 380–384 (in Japanese with English abstract).

[12] Fujita, R., Hirata, N. and Fujita, Y. Peroral Cholecystoscopy. *Endoscopy* 1989; **21**: 378–380.

[13] Foerster, E.C., Schneider, M.U., Matek, W. *et al.* Transpapillary cholecystoscopy. *Endoscopy* 1989; **21**: 381–383.

[14] Soda, K., Shitou, K., Yoshida, Y. *et al.* Peroral cholangioscopy using a new fine-caliber flexible scope for detailed examination without papillotomy. *Gastrointestinal Endosc.* 1996; **43**: 233–238.

[15] Igarashi, Y., Ishiguro, J., Anzai, T. *et al.* Endoscopic study of biliary carcinaoma by peroral cholangioscopy. *Stomach and Intestine* 1994; **29**: 777–784 (in Japanese with English abstract).

[16] Shinohara, Y., Fukuda, S., Takeda, K. *et al.* Experience with percutaneous electronic choledochoscopy. *Digestive Endoscopy* 1995; **7**: 150–154.

[17] Sato, M., Maetani, I., Ohashi, S. *et al.* Relationship between percutaneous transhepatic cholangioscopy findings and pattern of carcinaomatous spread in the bile duct. *Diagnostic and Therapeutic Endoscopy* 1994; **1**: 45–50.

